# Better palliative care for people with a dementia: summary of interdisciplinary workshop highlighting current gaps and recommendations for future research

**DOI:** 10.1186/s12904-017-0221-0

**Published:** 2017-07-14

**Authors:** Siobhán Fox, Carol FitzGerald, Karen Harrison Dening, Kate Irving, W. George Kernohan, Adrian Treloar, David Oliver, Suzanne Guerin, Suzanne Timmons

**Affiliations:** 1 Centre for Gerontology and Rehabilitation, School of Medicine, University College Cork, The Bungalow, Block 13, St Finbarr’s Hospital, Douglas road, Cork, T21XH60 Republic of Ireland; 2 Research & Evaluation Dementia UK, London, UK; 3 Nursing and Human Sciences, Dublin City University, Dublin, Ireland; 4 Institute of Nursing and Health Research, Ulster University, Co., Antrim, UK; 5 Old Age Psychiatry, Oxleas NHS Trust, London, UK; 6 Tizard Centre, University of Kent, Canterbury, UK; 7 EAPC Board Member, European Association of Palliative Care, Vilvoorde, Belgium; 8 Research Design & Analysis, School of Psychology, University College Dublin, Dublin, Ireland

**Keywords:** Dementia, Neurodegenerative diseases, Interdisciplinary research, Research priorities, Advance care planning, Personhood, Care at home

## Abstract

**Background:**

Dementia is the most common neurological disorder worldwide and is a life-limiting condition, but very often is not recognised as such. People with dementia, and their carers, have been shown to have palliative care needs equal in extent to those of cancer patients. However, many people with advanced dementia are not routinely being assessed to determine their palliative care needs, and it is not clear why this is so.

**Main body:**

An interdisciplinary workshop on “Palliative Care in Neurodegeneration, with a focus on Dementia”, was held in Cork, Ireland, in May 2016. The key aim of this workshop was to discuss the evidence base for palliative care for people with dementia, to identify ‘gaps’ for clinical research, and to make recommendations for interdisciplinary research practice. To lead the discussion throughout the day a multidisciplinary panel of expert speakers were brought together, including both researchers and clinicians from across Ireland and the UK. Targeted invitations were sent to attendees ensuring all key stakeholders were present to contribute to discussions. In total, 49 experts representing 17 different academic and practice settings, attended.

Key topics for discussion were pre-selected based on previously identified research priorities (e.g. James Lind Alliance) and stakeholder input. Key discussion topics included: i. Advance Care Planning for people with Dementia; ii. Personhood in End-of-life Dementia care; iii. Topics in the care of advanced dementia at home. These topics were used as a starting point, and the ethos of the workshop was that the attendees could stimulate discussion and debate in any relevant area, not just the key topics, summarised under iv. Other priorities.

**Conclusions:**

The care experienced by people with dementia and their families has the potential to be improved; palliative care frameworks may have much to offer in this endeavour. However, a solid evidence base is required to translate palliative care into practice in the context of dementia. This paper presents suggested research priorities as a starting point to build this evidence base. An interdisciplinary approach to research and priority setting is essential to develop actionable knowledge in this area.

## Background

Dementia causes impairment of memory, problem-solving and communication, and in advanced disease, the ability to perform everyday tasks [1]. Dementia is one of the major causes of disability and dependency among older people and it is not a normal part of ageing. In the United Kingdom and Wales, dementia is the leading cause of death [2]. Worldwide, 47.5 million people have dementia and there are 7.7 million new cases every year [3]. There are at least 48,000 people in the Republic of Ireland living with dementia; given the ageing population, this number is expected to increase to about 150,000 by 2046 [4]. While recent population-based research suggests that the prevalence rate of dementia in older people may actually be declining [5], due partly to improved healthcare, the number of people affected by dementia directly or indirectly continues to rise as the population ages and the number at risk rises.

There is a significant need to increase and expand the research base for palliative and end-of-life care, in recognition of emerging global priorities [6], including moving beyond cancer to examine other chronic diseases such as dementia [7]. Dementia is a life-limiting condition, but very often is not recognised as a terminal illness. People with dementia, and their carers, have been shown to have palliative care needs equal to those of cancer patients [8]. A palliative care approach is also favoured by informal caregivers [9].

Palliative care can be defined as: “an approach to care that improves the quality-of-life of patients and their families facing problems associated with life-threatening illness, through the prevention and relief of suffering by means of early identification and impeccable assessment and treatment of pain and other problems, physical, psychosocial and spiritual” [10]. This broad definition covers both i) generalist palliative care (approach which involves all healthcare workers practicing palliative care principles as a core skill, supplemented by some healthcare workers who are not engaged full time in palliative care, but have had additional training and experience in palliative care); and ii) Specialist Palliative Care services whose core activity is the provision of palliative care to individuals with more complex and demanding care needs [11].

Recent international reviews have highlighted the importance of palliative care in neurodegeneration [12–14]. The Irish National Dementia Strategy placed a particular focus on palliative care [15]. However, it is difficult to enact policy as the evidence base for the value of palliative care for people with dementia is still lacking and many people with advanced dementia are still not routinely being provided with palliative care in practice. Providing high quality palliative care for people with dementia presents unique challenges, for example the person’s inability to verbally express preferences for their care as the illness progresses, and the fact that the end-of-life phase may be long and difficult to identify [16]. Research is also hindered by the lack of agreed outcome measures, and the challenge of adapting existing tools for use with someone with advanced dementia who is verbally non-communicative [17]. Assessment of symptoms can be further confounded by the presence of concurrent illnesses.

In recognition of the importance of this challenge, the international research community has called for more clinically-relevant, collaborative, and strategic approaches to palliative care research [18–22]. While many disciplines have recognised the importance of research in palliative care for neurodegeneration individually, a problem is that researchers are tackling the problem from different perspectives, theoretical frameworks, and using diverse methodologies; these are complementary but require a platform for discussion, debate and collaboration. Furthermore, this discussion needs to be interdisciplinary, and include academics, practitioners and service-users, as one discipline alone cannot manage the complex physical, psychological, social, and ethical problems in palliative care for people with dementia. It is important that priorities for future research are set so that questions pertinent to dementia and palliative care in Ireland could be addressed effectively by researchers of all relevant disciplines, to enable a strong evidence base to be developed.

## Main text

### Planning the workshop

A consortium was established, representing two universities and five non-profit organisations for dementia and palliative care. The goal of the consortium was to plan an interdisciplinary workshop to explore the theme: “Palliative Care in Neurodegeneration with a focus on Dementia: Addressing complex questions through interdisciplinary research and reflection.” The aim of the workshop was to bring experts together from different disciplines to discuss this theme, to enhance cross-discipline learning, and to identify and discuss research gaps, priorities and methodologies in palliative care in neurodegeneration. There are other examples of using a similar approach to identify research priorities in palliative care (e.g. Stevinson, Preston, & Todd [23]; Jones et al. [24]).

The consortium members identified a long-list of key priority areas for the workshop through review of existing priority setting exercises. Members then conferred within their own organisations (this included input from a wider stakeholder network of academics and researchers, clinicians, and people affected by dementia) and a final short-list with particular relevance to the Irish context was agreed by the consortium. Next, experts in the chosen priorities were identified by the consortium and invited to the workshop. Five invited speakers presented at the workshop.

### The workshop

In total, 49 experts attended the workshop, representing academics, researchers, and clinicians, from a range of relevant disciplines (see Table 1). All attendees were identified and targeted as leading experts in Ireland in either palliative care, neurodegeneration, or both, and attendance was on an invite only basis. There was also substantial Patient and Public Involvement in both the organisation and attendance at the event, including family carers of people with dementia and representatives from national voluntary and charitable organisations.

**Table 1:** Details of professional backgrounds of workshop delegates

Discipline	n
Nursing	11
Consultant Physician	8
*Palliative Medicine*	*4*
*Geriatrician*	*2*
*Neurologist*	*1*
*Old Age Psychiatrist*	*1*
Psychology	7
Voluntary Sector	7
Medical Researchers	4
Law	3
Family Carers	2
Pharmacy	1
General Practitioner	1
Neuroscience	1
Microsystems	1
Physical Sciences	1
ICT For Healthcare	1
Speech And Language Therapist	1

The workshop was highly participatory, and scheduled such that all delegates had ample opportunity to partake in discussions throughout the day. The workshop included five facilitated discussions. In these sessions, invited speakers gave a brief introduction to one of the pre-identified key themes, and then an independent, second expert facilitated the consequent discussion with the floor. A longer keynote presentation was delivered by a leading international expert. Two workshop consortium members independently recorded the core discussion points as they arose, and also gathered and collated anonymous written comments (each attendee received blank comment cards for each session). The discussion points and the comments were synopsised by an expert in an afternoon session with further brief discussion to clarify content and fidelity. The workshop closed with a facilitated question and answer session with a panel of six experts, three of whom had presented earlier.

### Outcomes

The purpose of this paper is to summarise some of the key research priorities and suggestions for future research in dementia palliative care, based on core discussion points which arose during the workshop. We have provided a general overview of a selection of these key topics against a brief background literature. We conclude with specific priorities for future research which are taken directly from discussions during the workshop. This paper is not intended as an exact summary of the proceedings on the day, however video recordings of the workshop presentations are available online.

### Discussion topics

#### Overview of research in neurodegenerative disease

There is an imperative for the development of research into the care of people with neurodegenerative disease, as at present there are no curative treatments, and the aim of care is to provide the best supportive and palliative care for these patients and their families. There have been several documents and discussions about the future of this research including the Priority Partnership Project in 2015, which was based on a wide consultation on the future priorities for research in palliative care, initiated by Marie Curie and facilitated by the James Lind Alliance [25]. Ten areas were prioritised, and of these, the following four have particular relevance to neurodegenerative disease: access to palliative care; Advance Care Planning; determination of patient needs; assessment and treatment of pain when communication is complex (see Table 2).

**Table 2:** Selection of research priorities set through the James Lind Alliance and revised for Ireland by All Ireland Institute of Hospice and Palliative Care (2015)

Priority research questions identified by James Lind Alliance and All Ireland Institute of Hospice and Palliative Care (2015)
How can access to palliative care services be improved for everyone regardless of where they are in the UK? James Lind Alliance #2
What are the benefits of Advance Care Planning and other approaches to listening to and incorporating patients’ preferences? Who should implement this and when? James Lind Alliance #3
What are the best ways to begin and deliver palliative care for patients with non-cancer diseases (such as COPD, heart failure, MND, AIDS, multiple sclerosis, Crohn’s disease and stroke)? James Lind Alliance #6 / AIIHPC #9
What are the best ways to assess and treat pain and discomfort in people at the end of life with communication and/or cognitive difficulties, perhaps due to motor neurone disease (MND), dementia, Parkinson’s disease, brain tumour (including glioblastoma) or head and neck cancer, for example? James Lind Alliance #10
Priority research questions identified in May 2016 workshop
What are the limits and potential of proxy, i.e. family carers, decision making?
How best to include people with dementia in research studies, to achieve useful and actionable outcomes?
What is the economic benefit, if any, of care at home services for dementia, and other neurodegenerative disease?
What are the factors that contribute to and build carer resilience in advanced dementia care?
How can assessment and support through video technology be utilised?
What are the most appropriate outcome measures to explore benefit (if any) of palliative care? These need to be validated in dementia, Parkinson’s disease, motor neuron disease, etc.
What are the optimal methods to effect change in staff behaviours concerning palliative care for their patients with dementia?
What is the optimal transferrable model of dementia palliative care?
What is the incidence of, and how can we limit, chemical restraint through inappropriate antipsychotic prescribing in advanced dementia?
What is the effect on quality of death and dying, of being transferred from an acute hospital to die at home?
How can recognition of need be improved among primary care and other healthcare workers of palliative care needs in their patients with dementia, and other neurodegenerative disease?

The longer list of suggested research topics is also relevant, specifically: the best way of providing palliative care to people with dementia; swallowing problems at end-of-life; drooling, which often accompanies reduced swallowing; assessment of distress in dementia; carer support and training for carers; continuity of care; understanding the person’s needs in neurological disease and dementia.

Within Europe, the Joint Programme - Neurodegenerative Disease Research (JPND) has been considering the research priorities and suggested the following: needs assessment, the identification of transitions along the pathway (such as the move to institutional care), and consideration of effective models across Europe [26]. Suggested priorities include quality improvement and research funding to establish effective strategies to achieve them. Specific priorities within these two related domains have been identified (Table 3). These areas may now be considered in greater depth and it is hoped that there will be opportunities for funding to look at these areas.

**Table 3:** JPND Palliative and End-Of-Life Care Research in Neurodegenerative Diseases Suggested Priorities

The following areas are suggested priorities in two related domains:
Improvement of Quality
1. Support for transnational networking, aiming for multi-professional engagement in palliative care research across EU
2. Co-ordination of best practices across EU member states 2.1.Working groups looking at developing evidence a. Advance Care Planning b. Cognitive impairment and challenges c. Effectiveness of education d. Primary care involvement in planning for palliative care e. Engagement with voluntary groups
Research Funding
3. Collaborative research, especially enhancing and using existing population and disease based longitudinal cohort studies 3.1 . Looking at triggers / transitions leading to changes in care
4. Support of research into identification of best practices for needs assessment 4.1 . Multi-method 4.2 . Interdisciplinary

A recent Consensus document on neurological palliative care has been produced and endorsed by the European Academy of Neurology and the European Association for Palliative Care [14]. This Consensus has suggested areas for development in the palliative care for all patients with chronic and progressive neurological disease, considering in particular: ensuring palliative care approach is included in overall care, communication and Advance Care Planning, symptom management, multidisciplinary team approach, family support, carer support, bereavement care, discussion of end-of-life care and the recognition of end-of-life care and the identification and use of triggers for palliative care [14]. Research into these areas would help to facilitate these developments and provide the evidence base that is so often missing. A Delphi Study on palliative care for people with dementia, produced as a White Paper from the EAPC [27], found that the areas for research that received the highest importance ratings were person-centred care, communication and shared decision making; optimal treatment of symptoms and providing comfort, setting care goals and advance planning.

Together, these documents suggest that the palliative care needs of people with neurodegenerative diseases, including dementia, requires more research and there needs to be a unified approach, linked to existing evidence, and at all levels – locally, nationally, across Europe, and across the world. Such an approach should be informed by regional priorities and may be guided using specific frameworks and models of care.

#### Frameworks for planning and conducting research in palliative care and dementia

Dementia is a devastating illness which can affect every one of us in some way. Most widely, we all know someone with dementia and its symptoms and might all aim to achieve prevention in our own lives; a smaller number of us provide support and care for those so affected (and ourselves may need support); and an even smaller number attempt to address these needs through research and practice development.

The life-long journey is fraught with difficulty: those affected by dementia experience pain, loss of appetite, poor swallow, general fear and agitation, relationship problems and mental illness, infections, pressure ulcers and communication difficulties. If so affected, we need substantial help with activities of daily living and we might suffer social stigma and even the side-effects of treatments. The journey is at once unique to each of us, yet we must navigate it together and make decisions at all levels about where to place our emphasis.

**Fig. 1:**
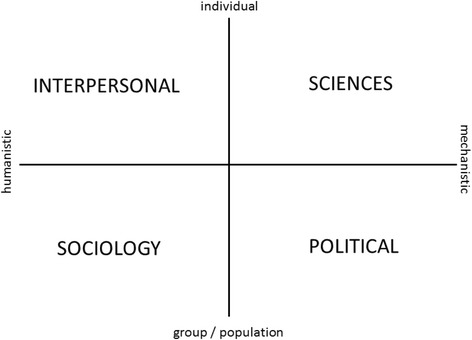
Showing the four quadrants of Hodges’ Health Career Model (1989) that provide a unique systematic way to think about research to inform holistic care

Two frameworks are offered to guide our thinking. First is the Health Career model devised by Hodges [28] which can be seen in Fig. 1. The model distinguishes four domains: sciences; political; sociology; and interpersonal, and challenges us to consider the potential to influence health outcomes from a range of viewpoints. From the mechanistic side, science and politics attempt to deal with cause and effect, costs and benefits, trade-offs and “hard” evidence to shape services. From the humanistic side come psychology, ethics, culture and sociology to address fear and stigma of illness, death and dying; addressing our relationships in support of one another. Hodges recognises the complexity of disease and, through his model, challenges modern thinking about how we address these challenging and interrelated symptoms of a complex disease. Interested readers are referred to this blog [29] for further reading. This model may provide a useful theoretical and conceptual framework for researching dementia and palliative care.

**Fig. 2:**
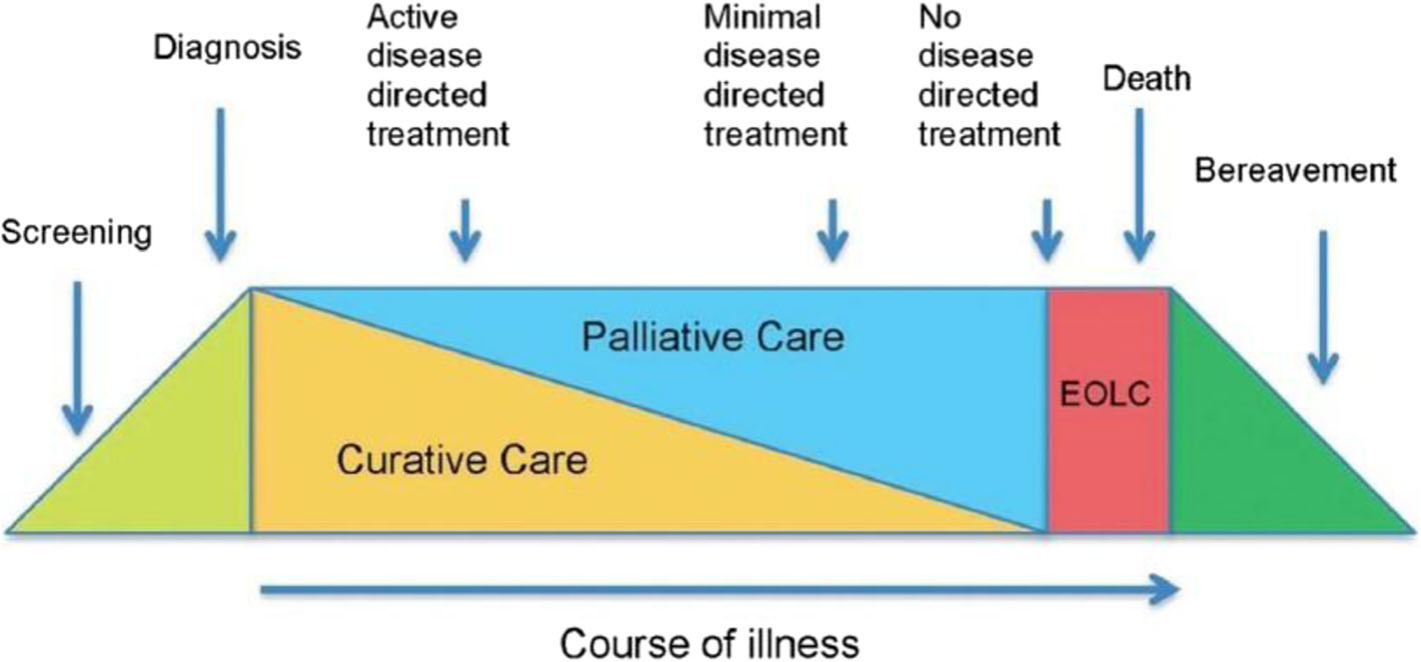
Showing the Palliative Care Continuum as one-dimensional journey from screening and diagnosis to end-of-life care. Evidence is required to inform practice in all segments (*coloured*)

**Fig. 3:**
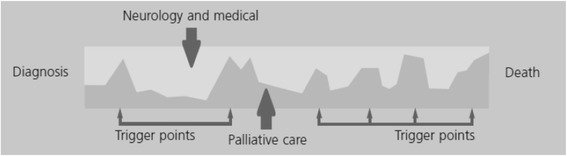
The model of dynamic involvement of palliative services based on trigger points (adapted from NHS England, End of life in long term neurological conditions: A framework for Implementation, pg.11)

The second framework is the more familiar schematic timeline, see Fig. 2. The palliative care continuum offers a somewhat more one-dimensional or simpler view of the journey from screening for disease in an otherwise healthy population, through diagnosis into a zone where elements of curative and palliative care combine to achieve quality-of-life, right up to (and including) death and (for those close by) bereavement support. The long course of the illness allows some potential to navigate the journey, address secondary prevention and consider rehabilitation models in order to achieve as good a quality-of-life as possible. There are other models of palliative care involvement, including a varying involvement, according to need, throughout the disease progression – as shown in Fig. 3. This model is of particular relevance in progressive neurological disease, such as dementia, where there are times of specific deterioration such as in feeding or breathing, but at other times the disease progression is slow.

Within the holistic remit of palliative care lie four primary components: the physical, social, spiritual and psychological. These frameworks aim to inform thinking, and highlight gaps in our knowledge where multi-professional, inter-disciplinary views, expertise and effort can be brought together to help make sense of complex issues in dementia. Specific research priorities have been identified (Table 2) and go some way to highlight the current unanswered questions. Hodges Health Career Model and the Palliative Care Continuum can help to ensure that the journey ahead is well-travelled. Future research could usefully explore the intersection of these two models.

#### Research priorities in Advance Care Planning for people with dementia

In the United Kingdom and Ireland, various policy documents have called for improvements in care for people with dementia towards the end-of-life by promoting the use of ‘Advance Care Planning’ [30–33]. In Ireland, pioneering legislation was introduced in 2015 in the form of the Assisted Decision Making (Capacity) Act [34], which provides the legal guidance to uphold the autonomy and dignity of the person with dementia, and may be an exemplar for other countries. It has been suggested that everyone should be encouraged to identify their needs, priorities and preferences for end-of-life care [30]. This may seem to be a challenge for those with mental capacity, but will be especially challenging for people with cognitive impairment and language deficits which reduces their ability to express their preferences. Autonomy in decision making depends upon consciousness of our past and future thoughts and actions in the same way as we are conscious of our present thoughts and actions [35]. However, as dementia progresses, in particular, the ability to consider future thoughts [36] and actions become compromised and this affects the capacity to make decisions [37].

*Proxy decision making.* Older people often trust loved ones to make healthcare decisions on their behalf [38] and want those decisions to be in keeping with their own wishes and preferences [39]. Family carers are assumed to know what these wishes and preferences would have been had the person with dementia not lost capacity [40] and professionals often rely on family members to predict and articulate these preferences with assumed accuracy [41]. However, research shows this assumption to be misplaced [37], with family carers often not able to accurately reflect the preferences of a person with dementia in the absence of prior discussions or a documented advance care plan [42]. Proxy decision making can be confounded as such decisions may be impossible to separate from the family carers’ own views and furthermore, where the family carer has supportive (or other) care needs of their own. Accordingly, the limits and potential of proxy decision making in the Irish context, require further clarification and research.

*Future research priorities for Advance Care Planning in Dementia.* Overall there is little evidence to support Advance Care Planning in dementia as a specific intervention. We need to test a feasible and acceptable Advance Care Planning intervention for families affected by dementia [43] and to test it over time. However, given the average life span of a person with dementia [44], this presents the researcher with considerable challenges. Funding for such a study that would recruit people with dementia from an early stage, when they are more likely to have the capacity to develop an Advance Care Plan; through to end-of-life, to be able to measure its effectiveness, may render it unfeasible in respect of normal funding time scales.

These long time scales assume that the only evidence for practice comes from long term prospective trails. Other forms of ethical decision-making can be informed by professional and personal experience of patients and family members. However, as noted above, there is scant evidence on the compatibility of the priorities and wishes of the family carer and the person with dementia, and if these change over time, converging or diverging, and if it is influenced by the progression of the disease or by transitions in care. Such evidence as exists suggests their perspectives differ greatly at the outset [42, 45] but, could an intervention be developed that works systemically with the whole family to develop a realistic, shared decision making approach? We know that families affected by dementia do benefit from early and ongoing practical and emotional support [46], but can this be extended to prepare them for potential changes and aid decision making in the context of the realities of care towards the end-of-life [47]? To do this, we need to develop a greater understanding of what factors influence the agreement or divergence of views, or how these issues are handled in skilled practice.

We also lack knowledge as to whether an Advance Care Planning intervention is a viable option for people in different stages of dementia. Often capacity assessments are not always carried out to consider specific decisions in respect of end-of-life care preferences so further study is warranted on how we can ensure people with dementia in the moderate to advanced stages of the illness are supported to engage in the decision making processes for their end-of-life care. We also need to establish the stability of these views over the dementia journey.

#### Research priorities in personhood in end-of-life dementia care

‘Person-centred care,' since its rise in popularity in the 1980’s, has become a catchphrase for good dementia care. However, while the phrase is central to policy and education on dementia, many people with dementia have not experienced improvements in care. The primary proponent of person-centred care in dementia, Tom Kitwood [48], made a very insightful statement in his book, Dementia Reconsidered:

*“It is conceivable that most of the advances that have been made in recent years might be obliterated, and that the state of affairs in 2010 might be as bad as it was in 1970, except that it would be varnished by eloquent mission statements, and masked by fine buildings and glossy brochures”* p.133.

If we are to ensure that person-centred care is more than a name-check in a mission statement, it is essential that we explore the meaning of personhood right along the spectrum of dementia to end-of-life care. Personhood is a standing or status that is bestowed on one human being, by others, in the context of relationship and social being. It implies recognition, respect and trust. It is a commitment on behalf of one to recognize the unique contribution of all human beings: primarily the person living with dementia, but also the family carer, the volunteer, the unqualified assistant and healthcare professionals [48].

For the research community there are many hurdles to surpass before we can realize this challenge. It can be difficult for ethics committees to accept the necessity of involving vulnerable people in research as co-researchers [49], a position which has led to a silence of the voice of people with dementia for too long. This position serves to reinforce the idea that people with dementia may not have a worthwhile contribution to make or that they are too vulnerable to require anything of them. Of course these concerns are to be taken seriously but the larger danger may well be the resulting lack of voice.

Assuming ethical permissions, there is an emerging but neophyte literature on the methods required to elicit useful data when people with dementia are taking part in research studies. As people with dementia are not in any way homogenous, the skills required are hugely varied not just from person to person, but from day to day and week to week, depending on context and many other factors we are yet to fully understand.

One example that explores the uniqueness of human response at the later stages of dementia is the AwareCare study [50]. They proposed that if care staff can be trained to identify signs of awareness this should support greater responsiveness and facilitate the expression of awareness. They found seven spontaneously occurring stimuli (e.g. someone nearby) and three introduced stimuli (e.g. call by name), with 14 response categories sub-divided into movement (eyes, face, head, arm and body) and sounds. Importantly, use of the tool led to relatives rating improvements in wellbeing and quality-of-life of the person with dementia.

There is a great need for creativity in research to generate knowledge that supports the translation of person-centred care not just as a watch-word for good care but as an illumination of how that may be practiced.

#### Research topics in the care of advanced dementia at home and in 24-h care

In Ireland and the United Kingdom, acute hospital care is under huge pressure with large overspends on unplanned emergency admissions. Older people occupy increasing numbers of acute care beds, and most people with dementia present to the Emergency Department or and/or acute medical assessment unit in the last six months of life [51]. Good care at home may help avoid this, and the associated costs, as well as supporting good outcomes. Advanced dementia care at home has been piloted by Treloar et al. [52] and further described by the Kings Fund [53]. Data from studies have indicated substantial savings as a result of advanced dementia care at home. Sampson et at [51] found that care costs over the six months before death were higher in care homes or continuing care (£37,029) than for those living at home (£19,854). The Housing 21 Dementia Voice project in Westminster [54] reported that “over a 24-month period, it is estimated that the Dementia Voice Nurse service wholly or partly contributed to savings of £314,440 through the avoidance of hospital, nursing and residential home admission and the use of ambulance services”. Results from the Hope for Home study [52] indicated that total savings of home care compared with nursing home care for 14 patients was approximately £700,000 and that 57% of participants died in their own home. An audit of 23 patients cared for by the Greenwich Advanced Dementia service in 2009 found that, in total, these patients were cared for at home for 6205 days or approximately 886 weeks. Savings to local health and social care commissioners from these patients were estimated at between £200 and £350 per week, saving upwards of £177,200 to £310,100 for these patients. These savings are notional as the numbers of people using the service are too small to enable commissioners to release money from closing beds [53]. Using similar assumptions, the Greenwich Advanced Dementia Project estimates that it saved over £2 million caring for 100 patients. However, this data is “soft” and formal economic analysis of such services is very difficult. There is a real need for better quality economic data to complement patient-focused outcome data.

Despite the possible economic savings, supporting the care of people with advanced dementia at home is poorly understood and rarely prioritised by statutory services. Central to enabling care at home for a person with advanced dementia, is carer resilience. The START (STrAtegies for RelaTives) trial implemented a manualised intervention programme and aimed to improve carer coping strategies. The trial demonstrated reduced depression and anxiety in family carers of people with Dementia at 8 months and 2 years post intervention and also suggested savings [55].

Palliative care of a person with dementia at home also depends upon skilled healthcare, and expertise that enables competent professional advice to support carers in what they are doing. The principles of care of the Oxleas Advanced Dementia Service are good guiding principles, these are outlined in Table 4.

**Table 4:** Principles of care of the Oxleas Advanced Dementia Service

A core belief of the Oxleas Advanced Dementia Service is that anyone cared for at home with advanced dementia deserves care co-ordination and on-going support. The service combines mental and physical health expertise, to look competently after patients with advanced dementia living at home and to:
• Comprehensively assess and plan ahead;
• Co-ordinate care;
• Respond quickly when needs are changing;
• Establish a palliative care framework with a focus on maximising quality-of-life, helping to avoid or shorten unnecessary and traumatic hospital admissions, treatments and investigations, and replace them with home care whenever possible;
• Offer excellent care towards the end-of-life;
• Relieve the carer from having to navigate alone within a complex care system while grieving.

#### Other research priorities

In addition to the aforementioned themes, there were a number of recurring issues raised during discussion sessions during the workshop; these are discussed briefly in the following paragraphs and summarised in Table 2.

i. Research design, including the choice of appropriate methodologies, can be challenging in palliative care and dementia. By nature, large scale trials and longitudinal studies will be difficult and may not always be feasible. It is also critical to identify the best ways to capture the potential benefit of Advance Care Planning in palliative care and dementia. A research priority must be the identification and validation of appropriate outcome measures to explore benefit (if any) of palliative care. It was agreed that this still-emerging research area would benefit from smaller scale studies in the short-term, including: quality improvement studies, smaller pilot studies, and observational studies to better inform interventions in future trials. This aligns with the recommendations of the Medical Research Council (MRC) framework for the evaluation of complex health interventions [56]. The MRC framework was developed in light of the limitations of randomised control trials, mainly limited contextual data, and outlines the steps for process evaluation, i.e. methods to assess fidelity and quality of implementation, clarify causal mechanisms and identify contextual factors associated with variation in outcome.
ii. There is a research gap concerning our understanding of the lived experience of the person with advanced dementia. In this context Public and Patient Involvement (PPI) in research is critical. However, it is important that PPI is not incorporated as a token exercise, but rather researchers must aim to achieve useful and actionable outcomes and goals through patient and public participation in research. It is essential that people with advanced dementia are also included in research. For this to happen, innovative research methods must be utilised, as many people living with dementia at this advanced stage will be verbally non-communicative.
iii. Palliative care for dementia, and neurodegeneration has been supported in policy for some years, however in practice this is a new area for many healthcare staff and there is a need for it to be actioned in routine practice across disciplines. Therefore, research needs to investigate the optimal methods to change healthcare workers’ behaviours concerning palliative care for their patients with dementia. There are various recognised methods, some may be ethically questionable, such as financially incentivising nurses and other healthcare staff. A better course may be to look at implementing education programmes, and critically assess the sustainability of change following an education intervention. These programmes might include methods to help staff to get to know the person with dementia better, to improve quality of care, etc. Overall, research is needed to investigate which methods are the best way to sustain positive changes in staff behaviours for the long-term.
iv. Another priority is to develop useful and transferrable models of best care. In developing these, the key questions are: how to best integrate palliative care and dementia care, and identification of the facilitators and barriers to such integration; how to integrate care not only across disciplines but also sectors, including acute, community, residential care; and determining the existing access to specialist services for people with dementia. A small number of existing clinics have pioneered models of palliative care for dementia or other neurodegenerative illnesses, and these can serve as exemplary models of excellence. Learning from existing models that are performing well may be done through a cross-case analysis to identify the core principles and practices that are happening at each site, mapping across the models to look at the commonalities and differences and build a taxonomy of that model. Thus (as above) more conceptual research is needed, in addition to large scale trials and studies. In any model, cost effectiveness is critical, but it is impossible to accurately measure cost effectiveness unless the model of care is properly described. The development of these frameworks would be highly useful as they could be subsequently replicated in multiple sites.

v. Other topics that arose at this workshop included “chemical restraint” and the issue of inappropriate antipsychotic prescribing; dying at home, particularly transferring people at end-of-life from an acute hospital setting to die at home, and the effect of this on quality of death and dying; palliative care in primary care; improving staff and carers’ recognition of need (i.e. if a need is not recognised by others, it will never be addressed); the potential use of technology to assist in assessment where communication is limited, and in supporting care provision; exploration of potential conflicts in the views of the person with dementia, their family and healthcare workers towards end-of-life. The considered application of frameworks (such as Hodges Model) may provide a useful mapping framework for priority setting and enable other areas requiring attention to be highlighted.

## Conclusions

The care experienced by people with dementia and their families has the potential to be improved through using palliative care frameworks. However, a solid evidence base is required to inform how to achieve such improvements. As a relatively new field, there are significant methodological and content areas where research is needed. An expert consortium has highlighted priorities for future research (Table 2). Integrated care may improve outcomes, notably quality-of-life, for people with dementia [57], hence an interdisciplinary approach to research and priority setting is essential to further actionable knowledge in this area. It is also imperative that there needs to be a unified approach at all levels – nationally, across Europe, and across the world.

This paper summarises key topics in dementia palliative care, based in part on a consensus workshop, and the research priorities discussed here were not identified through systematic or empirical research studies. Further, the priorities were discussed primarily with relevance to the Irish context, and while most are common to international dementia research, there may be country-specific priorities owing to unique cultures, different healthcare systems, different state of current research, etc. However notable strengths of this paper, and the workshop which stimulated its development, are that the consensus group included targeted national and international experts from a variety of academic and professional disciplines, and had substantial Patient and Public Involvement. A literature review was also performed to place the research priorities discussed into context of international research literature.

We have highlighted some of the research priorities for palliative care and neurodegeneration, as discussed by a consortium of multidisciplinary experts. We have also suggested two models or frameworks that may be useful in mapping out topics to guide research in palliative care for people with dementia and continue to prompt further questions.

## Abbreviations

EAPC: European Association of Palliative Care
JPND: Joint Programme - Neurodegenerative Disease Research
MRC: Medical Research Council
PPI: Patient and Public Involvement
